# Shared perceptions of flavored cigarette pack design among young adults who smoke in Mexico and the Philippines

**DOI:** 10.18332/tid/168376

**Published:** 2023-07-26

**Authors:** Graziele Grilo, Jennifer L. Brown, Joanna E. Cohen, Katherine Clegg Smith

**Affiliations:** 1 Institute for Global Tobacco Control, Department of Health, Behavior and Society, Johns Hopkins Bloomberg School of Public Health, Baltimore, United States; 2 Department of Health, Behavior and Society, Johns Hopkins Bloomberg School of Public Health, Baltimore, United States

**Keywords:** tobacco packaging regulations, flavored cigarettes, young adults

## Abstract

**INTRODUCTION:**

Tobacco industry documents reveal companies’ knowledge of a similar young adult market across countries in terms of attitudes and lifestyle aspirations. Some tobacco companies, therefore, use similar marketing approaches across different jurisdictions. We examined young adults’ perceptions of flavored cigarette packs, including those containing flavor capsules, in Mexico and the Philippines.

**METHODS:**

We conducted a secondary analysis of five focus groups held in Mexico and four in the Philippines with young adults who smoke (aged 18–24 years), separated by gender, in which participants interacted with cigarette packs purchased locally. Transcribed and translated data were thematically analyzed and compared between countries.

**RESULTS:**

Three major themes were identified: 1) Flavor capsules cigarettes are recognizable via pack design through imagery on the pack that is understood to signify capsules; 2) Colors signal flavor and make the pack attractive; and 3) Young adults who smoke identify the target audience for these products as young people and those who are beginning to smoke.

**CONCLUSIONS:**

Young adults who smoke in Mexico and the Philippines interpreted flavored cigarette pack design similarly and thought that young people are the main audience for these products. This suggests a successful marketing approach creating shared perceptions of flavored cigarette packs in different world regions. It is likely that similar tactics are used in other countries around the world. Therefore, jurisdictions might use evidence from other jurisdictions to support the implementation of evidence-based tobacco control policies. These findings also support the implementation of plain and standardized packaging and flavor bans that would also limit product innovation such as capsules.

## INTRODUCTION

Transnational tobacco companies (TTCs) have aggressively invested in increasing tobacco sales in low- and middle-income countries (LMICs)^1^. Reviews of tobacco industry documents uncover extensive market research done by TTCs to develop marketing strategies to promote tobacco products in different countries^2-5^. These reviews illuminate TTCs’ extensive knowledge of young people and people who have recently initiated smoking as potential consumers^2-4,6,7^, who hold common attitudes, values, and lifestyle aspirations across geographical regions^3^. As one example, research conducted by Philip Morris between 1985 and 1994 found an increased presence of Western values and culture in Asian countries, helping to shape their global marketing approach for Marlboro^3^. Philip Morris, therefore, largely used the same marketing strategies across countries to promote Marlboro cigarettes^3^.

Packaging is a key marketing tool used by the tobacco industry to promote their products^8^. Through packaging design features and appeals (e.g. color, pack shape, imagery on pack), TTCs target specific consumer groups^8^, associate their brand with core values held by young adults^9^, and influence perceptions of harm, appeal and expectations about the product^8^. Research finds similar appeals being used on cigarette packs sold in different countries, such as claims of less smoke smell^10^, feminine appeal^11^, the use of less harm descriptors such as ‘natural’^12^, and the use of English text in non-Anglophone countries^13^. Vibrant colors and imagery of small spheres on packaging are often used on flavored and flavor capsule cigarette packs, which appeal to youth and are associated with smoking initiation^14,15^.

Mexico and the Philippines were selected as prime locations to conduct research on consumer perceptions of flavored cigarettes because they are both important markets for flavored cigarettes, specifically menthol cigarettes in the Philippines, and flavor capsule cigarettes in Mexico. Both countries are also home to large numbers of people who smoke. Current adult cigarette smoking prevalence in Mexico is 17.9% and in the Philippines it is 22.5%^16^. Both countries ban tobacco advertising on domestic television and radio and on the internet^17,18^, making marketing on cigarette packaging particularly salient.

We conducted studies in Mexico (2018) and the Philippines (2019), to examine consumer perceptions of flavored cigarette products among young adults who smoke to better understand how they interpret packaging design elements. Here, we focus on describing the similarities and differences in how young adults in two very different markets discern cigarette product properties based on the packaging. Our research fills a gap in the literature where there is a dearth of research that explores and compares consumer perceptions of similarly packaged cigarette products across jurisdictions, given that we know some tobacco companies approach marketing in different localities similarly.

We re-engaged with the data collected in Mexico and the Philippines and conducted a secondary analysis to explore the following research questions: 1) In Mexico and the Philippines, how do young adults who smoke interpret packaging design features communicating flavor, mainly flavor capsules?; and 2) What is the perceived audience for flavor capsule cigarettes and how is it similar or different between Mexico and the Philippines? Our goal is that findings might inform tobacco control interventions.

## METHODS

We conducted a secondary analysis of nine focus group (FG) discussions, stratified by gender and socioeconomic status (SES), based on public data sources about the SES level of the neighborhood in which the participants lives rather than individual-level SES, with young adults who smoke (aged 18–24 years) held in Mexico City, Mexico (November 2018) and in Metro Manila, Philippines (March 2019) (Table 1). Although two separate studies, the FG procedures and guide used in the Philippines were developed based on protocols from the Mexico study and one of the research team members (JC) was involved in both studies. In Mexico, people who smoke were defined as individuals who smoked at least 100 cigarettes in their lifetime and smoked cigarettes in the past week; in the Philippines, people who smoke were defined as individuals who smoked at least 100 cigarettes in their lifetime and currently smoke every day/some days. The overall primary purpose of both studies was to identify key cigarette pack design features that are attractive to young people. This secondary analysis focused on exploring perceptions of pack features that communicate flavor among young adults who smoke and comparing them across two countries.

**Table 1:** Focus group participant characteristics, Mexico and Philippines, Focus Group Discussions, 2018–2019 (N=48)

*Focus group*	*Number of participants*	*Country*	*Gender*	*SES*
1	4	Mexico	Women	Low
2	4	Mexico	Women	Mid/High
3	2	Mexico	Men	Low
4	3	Mexico	Men	Low
5	4	Mexico	Men	Mid/High
6	7	Philippines	Women	Low
7	8	Philippines	Women	High
8	8	Philippines	Men	Low
9	8	Philippines	Men	High

In both countries, participants interacted with a sample of locally purchased cigarette packs and engaged in discussions around perceptions of pack design and subjective evaluations of pack attractiveness. In Mexico, 23 packs were shown to participants, including 13 flavored cigarette variants (of these, 10 were flavor capsule variants). In the Philippines, a sample of 26 packs were shown to participants, including 15 flavored cigarette variants (of these, 10 were flavor capsule variants). All packs except one in Mexico and three in the Philippines were manufactured by TTCs, including Philip Morris International (Philippines and Mexico), Japan Tobacco International (Philippines and Mexico), British American Tobacco (Mexico), and KT&G (Philippines). Participant recruitment and focus group procedures have been published elsewhere^19,20^. Both studies were approved by an ethics committee in their respective country and in the US.

### Data analysis

Two authors (GG and JB) read the de-identified and raw English translations of the transcripts from both countries to familiarize themselves and took notes on their impressions and recurrent themes. After reviewing and discussing their notes, they developed a single codebook to be applied to all data based on the research questions and emerging themes. The researchers explored participants’ responses to packaging design related to flavor and flavor capsule cigarettes, including flavor imagery and descriptors and other ways they were communicated through packaging design. Additionally, they examined participants’ views about the perceived audience for flavored cigarettes. The coding process was facilitated by MAXQDA Analytics Pro 2018. In terms of coding processes, first, two transcripts were independently double-coded with the purpose of refining the codebook; then coding differences were discussed and consensus achieved after which changes were made to the codebook. After incorporating the changes, one more transcript was double-coded in which no major differences were identified. Each researcher then independently coded three transcripts. Following coding, data were thematically analyzed, and summaries were created using illustrative quotes. Findings were compared between countries.

### Public involvement in the study

This study explored public attitudes (among young adults who smoke) towards flavored cigarettes. The goal is to center the experiences and attitudes of the targets of tobacco industry marketing tactics to develop global tobacco control policy initiatives appropriately and effectively. Data collection centers on generating perspectives of young adults who smoke in two low- and middle-income countries (Mexico and the Philippines), and tobacco control advocates from these countries were involved in discussions around the goals of the studies that generated data for this secondary analysis. These discussions had the primary goal of aligning research with tobacco control policy priorities for the respective country, ensuring that we are being culturally sensitive. Findings will be shared with in-country advocates, who will decide how to use them to inform their work.

## RESULTS

**Figure 1:**
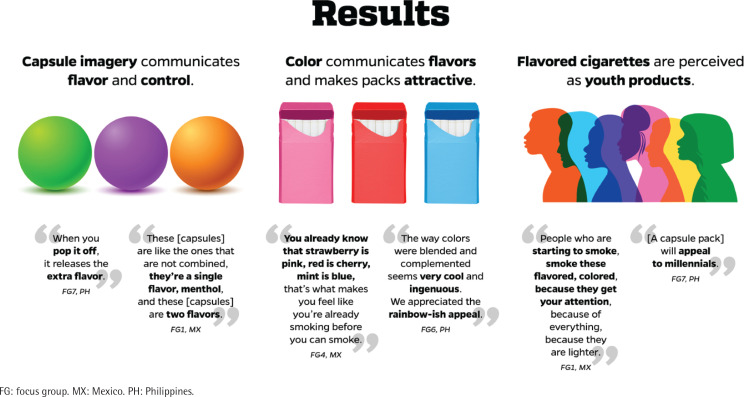
Summary of main findings, Mexico and Philippines, Focus Group Discussions, 2018–2019 (N=48)

**Figure 2:**
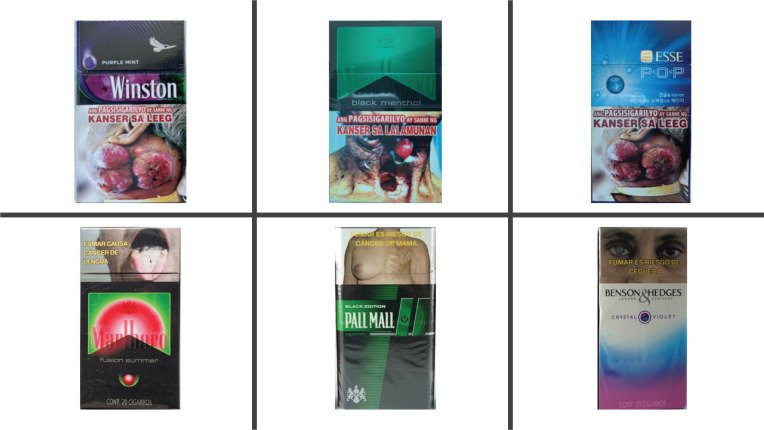
Example of packs from the Philippines (top) and Mexico (bottom) that participants interacted with. All packs except the Marlboro black menthol (Philippines) have some depiction of a flavor capsule on the pack – either in the form of a ball/circle of color or a ‘power button’. They also illustrate the different colors and flavor associations identified by the participants such as green for menthol and blue for mint, Mexico and Philippines, Focus Group Discussions, 2018–2019 (N=48)

Young adults who smoke in Mexico (MX) and the Philippines (PH) similarly described flavored cigarette pack features and presented comparable perceptions in terms of the main audience for these products. Participants easily identified flavored capsule cigarettes through imagery on packs, and they associated pack colors with specific flavors; moreover, imagery and colors increased their assessments of the attractiveness of the pack (Figures 1 and 2).

### Capsule imagery communicates flavor and control

Participants from seven of the nine FGs (except for FG5 and FG6) explicitly mentioned that they identified flavor capsules through the imagery on packs. The depicted capsule(s) were described as a ‘circle’, ‘dot’, ‘splash feature’, ‘bead’, and ‘pop’:

*‘The little dots, I recognize [them], it means … they come with a capsule.’* (FG3, MX)

*‘It’s still considered as menthol because of the 'splash' feature.’* (FG7, PH)

Flavor capsules were not only identified by the participants, but they also knew they would release the flavor once pressed, giving them the ability to ‘control’ their smoking experience:

*‘When you pop it off, it releases the extra flavor.’* (FG7, PH)

*‘Because it comes with, the one that has a capsule, a little circle; with that we can work it out.’* (FG4, MX)

*‘You can control it. It’s really cool.’* (FG8, PH)

Referring to the capsule imagery on the pack, participants in Mexico identified how many flavors a stick had, if it was one capsule with two flavors, or two capsules with one flavor each:

*‘These [capsules] are like the ones that are not combined, they're a single flavor, menthol, and these [capsules] are two flavors.’* (FG1, MX)

In the focus groups with Filipino participants, flavor capsules tended to be described as ‘cooling’ when smoked. In the focus groups in Mexico, flavor capsule cigarettes were described as ‘smoother’ than non-flavored cigarettes, which seemed to connote a somewhat different sensation compared with the cooling sensation; yet, both ‘cooling’ and ‘smoother’ were an indication that the harshness of the cigarette was reduced:

*‘The circle is like a candy ... Once it burst, it will be cooler.’* (FG8, PH)

*‘This one is much smoother than this one. I can't stand the smell of this one. And this one is much smoother and also has the capsule. It says that it's double burst because it has two capsules.’* (FG2, MX)

### Color communicates flavors and makes packs attractive

Participants across all FGs explained that they expected different cigarette flavors based on the pack color and flavor capsule color:

*‘We can also base on the color, since the color corresponds with the flavor most of the time.’* (FG7, PH)

*‘Going by the colors, you could even deduce that this is mint flavored.’* (FG5, MX)

*‘Most of the menthol cigarette colors are green, blue, and black.’* (FG8, PH)

*‘Like the flavorings that already have a predetermined color. You already know that strawberry is pink, red is cherry, mint is blue, that's what makes you feel like you're already smoking before you can smoke.’* (FG4, MX)

In addition to indicating a specific flavor, the colorfulness of a pack also seemed to evoke generalized or novel flavor expectations:

*‘For the colors, more than the taste, for the colors, the presentation [of the] Crystal Violet was new to me at one point, and it strikes me as a good flavor.’* (FG3, MX)

*‘There are a lot of colors, it looks like a candy.’* (FG8, PH)

Overall, participants indicated that colors increased the appeal of the cigarette pack, especially certain combinations, blended design, and vibrant colors:

*‘The way colors were blended and complemented seems very cool and ingenuous. We appreciated the rainbow-ish appeal.’* (FG6, PH)

*‘The pack seems elegant to me. And I especially like the color, and also the font, they're very attractive.’* (FG2, MX)

*‘The ones we deemed attractive are colorful.’* (FG7, PH)

*‘These are the ones I'd point out as like, more appealing, simply because they aren't a single color … They combine more of the colors. I'd define that as more eye-catching.’* (FG5, MX)

### Flavored cigarettes are perceived as youth products

Except for one male FG in Mexico (FG5) and one in the Philippines (FG9), participants shared the view that packs with flavor and flavor capsules are most commonly used by and designed for young people and those experimenting with smoking:

*‘[A capsule pack] will appeal to millennials.’* (FG7, PH)

*‘I go to the high school I used to attend, [and] most people smoke flavored [cigarettes]. … The kids, young people, the ones who are starting to smoke regularly, smoke one of these flavored ones and those who've been smoking longer smoke these [non-flavored cigarettes]. … So, I think that … people who are starting to smoke, smoke these flavored, colored, because they get your attention, because of everything, because they are lighter.’* (FG1, MX)

*‘The brand is really affordable, but the flavor makes it more appealing to the younger ones.’* (FG6, PH)

## DISCUSSION

Young adults who smoke in Mexico and the Philippines interpreted flavored cigarette pack design features similarly and agreed that younger people, like themselves, are the main audience for these products. The extensive similarity of the findings suggest that the standardized marketing approach used by TTCs has been successful in creating shared meaning and perceptions of flavored cigarettes through packaging among young adults in geographically dispersed regions of the world.

Flavored cigarettes and flavor capsule cigarettes were recognizable via packaging by the participants in this study across two different markets. This finding is consistent with findings from a systematic review that found high awareness of flavor capsule cigarettes among adolescents and younger adults^14^. It is possible that high awareness stems from exposure to flavor capsule cigarette packaging. Study participants, as people who smoke themselves, were able to easily identify packaging features, like color and imagery that communicate flavors; this is also consistent with past research^21^. Moreover, in the case of flavor capsule cigarettes, our results support that the tobacco industry has been successful in developing a product that young smokers in two regions of the world could recognize as being marketed towards them and their peers and younger people^22^. These findings highlight the use of packaging in the construction of a global product (i.e. flavor capsule cigarettes) by creating associations that persist across cultures.

In both countries, participants indicated that flavor capsule cigarettes appeal to people who are experimenting with smoking, newer to smoking, and occasionally smoke. This is consistent with other studies that conclude flavored cigarettes encourage experimentation^21,23^. Moreover, studies in the US have reported a shift in the age of smoking initiation from adolescence (aged 11–17 years) to young adulthood (aged 18–24 years), which might be partially explained by the increased marketing restrictions that decrease exposure among adolescents (especially via mass media channels) but not necessarily in young adults (for example, at the point-of-sale, in which price promotions are also employed)^24^. These marketing restrictions have contributed to changes in social norms around smoking, deglamorizing what once was part of adolescents’ identity formation^25^ and might now be more related to young adulthood^24^. The participants in this study also talked about the use of flavor capsule cigarettes in social settings, such as school and parties, with their peers. This could impact smoking behaviors among young people if there is acceptability and likeness by their peers, reinforcing social norms around smoking^26^ and contributing to their identity formation as people who smoke.

### Strengths and limitations

The central limitation of this study was that it consists of a secondary analysis of qualitative data from two different studies, which included different, yet commonly used definitions of smoking status. This design restricted investigators in terms of the content and focus of the transcripts, not allowing for further probing of participants. In terms of the data collection, it was sometimes challenging to recruit young adult smokers to participate in the FG discussions in Mexico, and as a result, two of the focus groups were very small. Additionally, these data are about five to six years old now, pre COVID-19 pandemic and the proliferation of electronic cigarettes, which may have contributed to shifts in tobacco and nicotine use behaviors. However, the flavor capsule cigarette market in both countries is still expanding^27-29^. For example, in Mexico, the cigarette pack continues to be a valuable communication channel filled with beautiful and creative design features, including ones that communicate flavor capsules^30^. Despite presenting data from only two countries, we feel that it is important that we found similar perceptions of flavor capsule cigarettes in two different regions of the world. Moreover, adolescents from both countries were able to tell that those products were marked towards them, influencing their smoking behavior and appealing to the persona they are trying to portray as they develop their identity in young adulthood. It is likely that similar marketing practices and perceptions of these practices are persistent in jurisdictions beyond Mexico and Philippines. The strengths of this study include the use of similar discussion guides and procedures and inclusion of participants of the same age group. Also, research that is conducted with the target audience of the marketing we intend to target through policy and communication interventions (in this case, young adults who smoke) increases the face validity of our findings and making them directly applicable to policy and practice.

### Implications for policy and practice

Findings from this study that demonstrate shared perceptions of flavored packaging design across two jurisdictions might help to strengthen the argument that robust evidence from one or a few jurisdictions could be considered as supporting evidence for implementation of evidence-based tobacco control policies elsewhere, such as those required by the WHO Framework Convention on Tobacco Control^3^. It is not uncommon that jurisdictions look to other places to support policy action. For example, the comprehensive and rigorous evidence on plain and standardized packaging from Australia and the United Kingdom has supported the implementation of plain and standardized packaging in several countries, including Thailand and Uruguay. These countries relied mostly on evidence beyond their borders and did not require a country-specific legislative impact assessment to support adoption of their regulations^31^. While local evidence is needed in unique instances, being able to generalize study findings to other jurisdictions can be valuable when research is under resourced or when time to conduct rigorous research is limited.

These findings can also support efforts calling for implementation of plain and standardized packaging (including the cigarette stick), bans on product display at point-of-sale, and a ban on flavored cigarettes (including devices like capsules that deliver flavor). Jurisdictions outside Mexico City and Metro Manila may heed the findings of this study as a warning that encourages policies to address flavored cigarettes and the mechanisms used to deliver flavor to consumers. As part of tobacco control interventions aimed at denormalization of smoking among young adults, it is essential to curb the capacity for cigarettes to be made more palatable by flavors, and with elements that reinforce vibrancy, control and attractiveness that are so valuable to people at young age, as reported by the participants of this study. Innovative tobacco control policies, such as the single pack presentation in Uruguay, can also hamper the cigarette pack from being an effective marketing channel^32^.

## CONCLUSIONS

Transnational tobacco companies have employed similar marketing strategies via packaging design when promoting flavored cigarettes, and specifically flavor capsule cigarettes, in Mexico and the Philippines, two countries in different regions of the world. It is likely that these strategies are being used in other places as well to target young adults, who find flavored cigarettes particularly appealing. Moreover, flavored cigarettes might be contributing to smoking initiation among young adults as they are in the process of developing their identity. When resources are limited, jurisdictions might consider using research findings from other places to support evidence-based tobacco control policies, as those in the WHO Framework Convention on Tobacco Control. Moreover, our findings support the implementation of plain and standardized packaging, product display ban at the point-of-sale and bans on flavored tobacco, including flavor capsules and other filter innovations, in Mexico and the Philippines.

## Data Availability

The data supporting this research are available from the authors on reasonable request.
